# Pathogenic PHIP Variants are Variably Associated With CAKUT

**DOI:** 10.1016/j.ekir.2024.05.024

**Published:** 2024-05-27

**Authors:** Jonathan de Fallois, Tobias Sieckmann, Ria Schönauer, Friederike Petzold, Johannes Münch, Melissa Pauly, Georgia Vasileiou, Christin Findeisen, Antje Kampmeier, Alma Kuechler, André Reis, Eva Decker, Carsten Bergmann, Konrad Platzer, Velibor Tasic, Karin Michaela Kirschner, Shirlee Shril, Friedhelm Hildebrandt, Wendy K. Chung, Jan Halbritter

**Affiliations:** 1 Division of Nephrology, Department of Internal Medicine, University of Leipzig Medical Center, Leipzig, Germany; 2 Institute of Translational Physiology, Charité Universitätsmedizin Berlin, Berlin, Germany; 3 Department of Nephrology and Medical Intensive Care, Charité Universitätsmedizin Berlin, Berlin, Germany; 4 Institute of Human Genetics, Universitätsklinikum Erlangen, Friedrich-Alexander-Universität Erlangen-Nürnberg, Erlangen, Germany; 5 Institute of Human Genetics, University Hospital Essen, University of Duisburg-Essen, Essen, Germany; 6 Medizinische Genetik Mainz, Limbach Genetics, Mainz, Germany; 7 Institute of Human Genetics, University of Leipzig Medical Center, Leipzig, Germany; 8 Faculty of Medicine, University Ss. Cyril and Methodius, Skopje, North Macedonia; 9 Department of Pediatrics, Boston Children's Hospital, Harvard Medical School, Boston, Massachusetts, USA

**Keywords:** CAKUT, Chung-Jansen syndrome (CHUJANS), PHIP

## Abstract

**Introduction:**

Congenital anomalies of the kidney and urinary tract (CAKUT) represent the most common cause of chronic kidney disease in children. Although only 20% of cases can be genetically explained, the majority remain without an identified underlying etiology. The neurodevelopmental disorder Chung-Jansen syndrome (CHUJANS) is caused by haploinsufficiency of Pleckstrin homology domain-interacting protein (PHIP) and was previously associated with genital malformations. Anecdotal coincidence of CHUJANS and CAKUT prompted us to investigate whether urorenal malformations are part of the phenotypic spectrum of CHUJANS.

**Methods:**

Analysis of existing CHUJANS and CAKUT cohorts, consulting matchmaking platforms, and systematic literature review to look for additional patients with both CHUJANS and CAKUT. Prenatal expression studies in murine and human renal tissues to investigate the role for PHIP in kidney development.

**Results:**

We identified 4 novel and 8 published cases, indicating variable expressivity with a urorenogenital trait frequency of 5% to 35%. The prenatal expression studies supported a role for PHIP in normal kidney and urinary tract development.

**Conclusion:**

Pathogenic *PHIP* gene variants should be considered as causative in patients with syndromal CAKUT. Conversely, patients with CHUJANS should be clinically evaluated for urorenogenital manifestations. Because neurodevelopmental disorders are often associated with kidney phenotypes, an interdisciplinary re-evaluation offers promise in identifying incompletely penetrant kidney associations and uncovering novel molecular mechanisms of disturbed nephrogenesis.

## Graphical abstract

**Figure:**
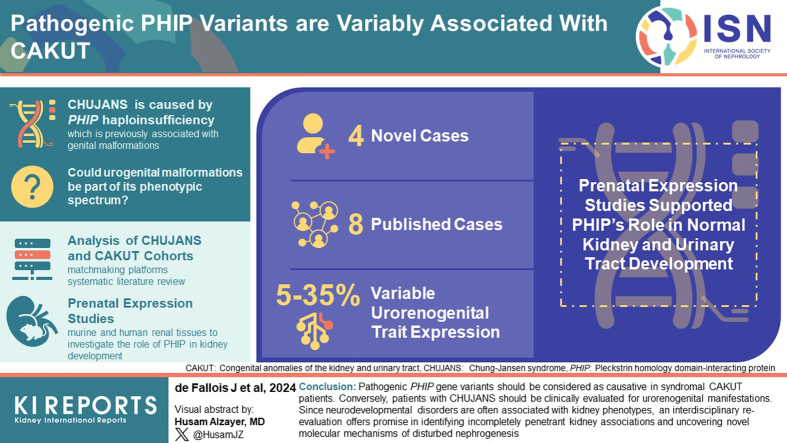

Disturbances during kidney and urinary tract development result in a wide spectrum of malformations collectively referred to as CAKUT.^1^^,^^2^ These malformations include kidney agenesis, hypoplasia, multiplex/duplex kidneys, multicystic dysplasia, ureteropelvic junction obstruction, ureterovesical junction obstruction, vesicoureteral reflux, posterior urethral valve disorders, and various other malformations.^3^

CAKUT represent the most common cause of pediatric chronic kidney disease and can progress to kidney failure over time.^2^^,^^4^, ^5^, ^6^ Monogenic causes account for ∼20% of CAKUT and an additional 5% to 10% of cases are attributed to larger copy number variations affecting functions of multiple genes.^7^ To date, >50 genes have been associated with CAKUT, a number that is steadily growing.^8^ CAKUT may present as an isolated, nonsyndromic condition, but is often part of a syndromic disease with multi-organ involvement.^1^^,^^2^ In syndromic diseases, such as neurodevelopmental disorders, urorenogenital phenotypes may only occur in a subset of patients as previously demonstrated for *ATN1*,^9^ *ROBO1*,^10^ *KIF4A*,^11^ and other associated genes. The exploration of a possible urorenogenital involvement in established congenital anomaly syndromes, notably neurodevelopmental disorders, may provide insights into molecular mechanisms of nephrogenesis and enable the discovery of novel CAKUT candidate genes.

CHUJANS (MIM# 617991) is a distinct neurodevelopmental disorders caused by heterozygous loss-of-function variants in *PHIP* (MIM ∗612870).^12^ *PHIP* encodes for 3 functional proteins through alternative splicing: the 902-amino acid C-terminus called PHIP1,^13^ the 1019-amino acid N-terminus called neuronal differentiation-related protein,^14^ and the full-length protein called DNA damage binding protein 1 and CUL4 (Cullin4)-associated factor 14 (DCAF14).^15^ The latter belongs to the DCAF protein family and acts as a substrate receptor in the ubiquitin ligase pathway,^16^^,^^17^ regulating and controlling the essential ubiquitination reaction.^18^ *PHIP* haploinsufficiency leads to complex clinical phenotypes characterized by developmental delay, intellectual disability, obesity, dysmorphic features, and cryptorchidism.^12^^,^^19^, ^20^, ^21^, ^22^, ^23^ Until recently, there was no established link between urorenal anomalies and this disorder. Prompted by the genetic diagnosis of CHUJANS in an adult patient with CAKUT-related KF (index patient), we hypothesized that pathogenic variants in *PHIP* might interfere with normal kidney development. In this study, we aimed to systematically investigate the frequency of CAKUT as a variable trait in patients with CHUJANS.

## Methods

### Patients

The index patient (ID1) and her family were recruited from the transplant unit at the University of Leipzig Medical Center. The legal guardian provided written informed consent, and the study was approved by the Institutional Review Board (IRB) at the University of Leipzig, Germany (IRB00001750; #402/16-ek) and by the IRBs at Columbia University New York and Boston Children`s Hospital, Boston, USA. To search for more cases, we used the GeneMatcher platform (https://genematcher.org)^24^ and systematically reviewed the literature for published cases of CHUJANS and CAKUT or complex genital malformations. Furthermore, we screened established cohorts of patients with CHUJANS for CAKUT spectrum at the Department of Medicine at Columbia University, New York (*n* = 61), and at the institute of Human Genetics at University Leipzig Medical Center, Leipzig (*n* = 4). Conversely, exome data from a cohort of unresolved CAKUT cases were screened for CHUJANS at Boston Children’s Hospital, Boston (848 families). For reverse phenotyping, we collected detailed clinical data of the newly identified patients using structured clinical questionnaires. All newly reported patients provided written informed consent for publication.

### Molecular Genetics

ID1 underwent whole-exome sequencing conducted from blood-derived DNA samples. Virtual panel analysis excluded competing variants in known genes associated with CAKUT and other hereditary nephropathies. Additionally, parental segregation analysis was done through direct Sanger sequencing to confirm de novo status. Whole-exome sequencing was also performed in ID2.1 and ID2.2, and the parenteral segregation was done by direct Sanger sequencing. The family of ID3 was studied using trio whole-exome sequencing. Copy number variation analysis was performed on the basis of high coverage next-generation sequencing data to the best of our knowledge. For ID4-11 next-generation sequencing-based clinical exome or gene panel testing were conducted as previously published.^19^^,^^21^^,^^22^^,^^25^ All variants are reported using the reference sequence NM_017934.7 and checked for existing entries in main population and patient databases (gnomADv4 [http://gnomad.broadinstitute.org/],^26^ ClinVar (last accessed on January 31, 2024, and HGMD version 2023.4).

### Immunostaining in Human Fetal Kidneys

To demonstrate the presence of PHIP in the developing human kidney, we performed immunostaining targeting PHIP with polyclonal rabbit anti-PHIP antibody (Cat# HPA019140, RRID:AB_2670073, Sigma-Aldrich, Darmstadt, Germany).^27^ We stained fetal human kidney sections from weeks 14 to 22 gestational age.

### Murine RNA In Situ Hybridization

Expression of *Phip* mRNA was assayed in the developing mouse kidney by in situ hybridization using RNAScope multiplex V2 Reagent Kit (ACD a biotechne brand, Newark, USA) according to the manufacturer’s instruction. In detail, embryonal mouse kidneys at Theiler stage (TS) TS20, TS22, TS24, and postnatal at day 3, 7, and 14 and after 11 weeks (adult) were stained for *Phip* mRNA using specific probe 1221641-C1 (ACD a biotechne brand, Newark, USA). To highlight the metanephric mesenchyme and developing glomeruli, mRNA of the podocyte marker Wilms Tumor 1 (Wt1) was assayed with probe 432711-C2 (ACD a biotechne brand, Newark, USA). Counterstaining on nuclei was performed with 4',6-diamidino-2-phenylindole. Tissue samples of embryos and postnatal animals were collected from C57Bl6 mice. The procedures were performed under the license T0063/20 issued by the local authorities (Landesamt für Gesundheit und Soziales Berlin, LAGeSo, Germany). After excision, kidneys were immersion fixed using Roti Histofix (Carl Roth, Karlsruhe, Germany) for at least 24 hours. After fixation, samples were paraffinized using ethanol solutions of increasing concentration followed by infiltration with paraffin. Quantification of PHIP signal was performed on 5 to 10 nonoverlapping images (depending on the size of the kidney), each representing 0.019 mm^2^ of the assayed kidney. To count *Phip* punctuae, the 8-bit image of the channel was subjected to automated thresholding, and the resultant particles were analyzed using ImageJ version 1.53.

## Results

### Clinical Characteristics and Genetic Analysis of the Index Patient

**Figure 1:**
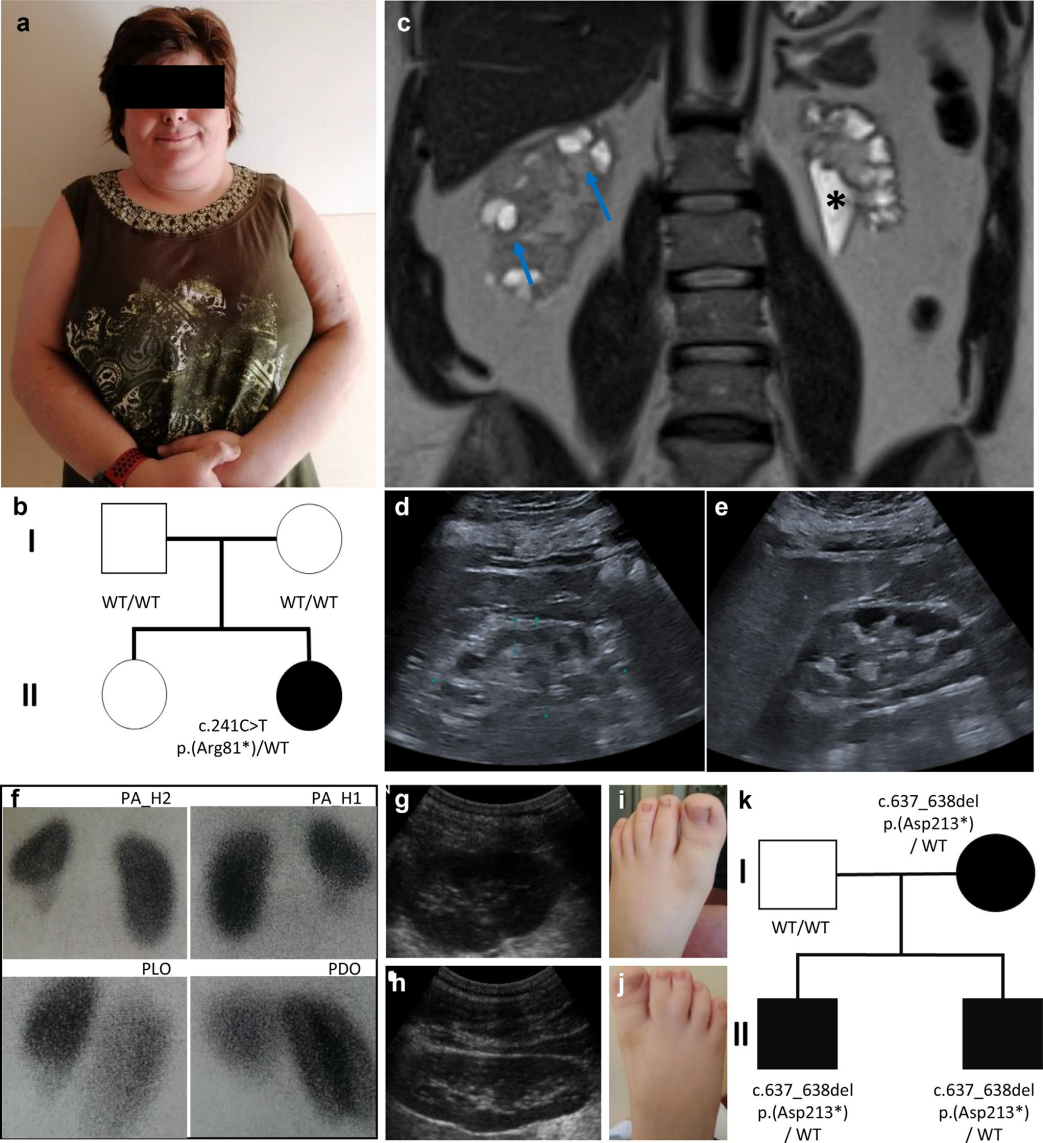
Selected clinical images and pedigrees. (a**–**e) Illustrates index patient (ID1) aged 42 years: (a) Stature and facial appearance, (b) pedigree, indicating de novo status, (c) MRI of the kidneys with significant narrowing of the parenchyma and enlarged renal calyces, probably expression of chronic urinary stasis grade III-IV° (∗). Cortical kidney cyst left up to 2 cm in diameter (blue arrows), ultrasound imaging of the left (c) and the right (e) kidney. (f–k) illustrates ID 2.1: 99mTc-DMSA scintigraphy of the kidneys (f), ultrasound imaging of the left (g) and right (h) kidney with unilateral kidney hypoplasia, (i–j) syndactyly, (k) and pedigree of the family indicates autosomal inheritance.

**Figure 2:**
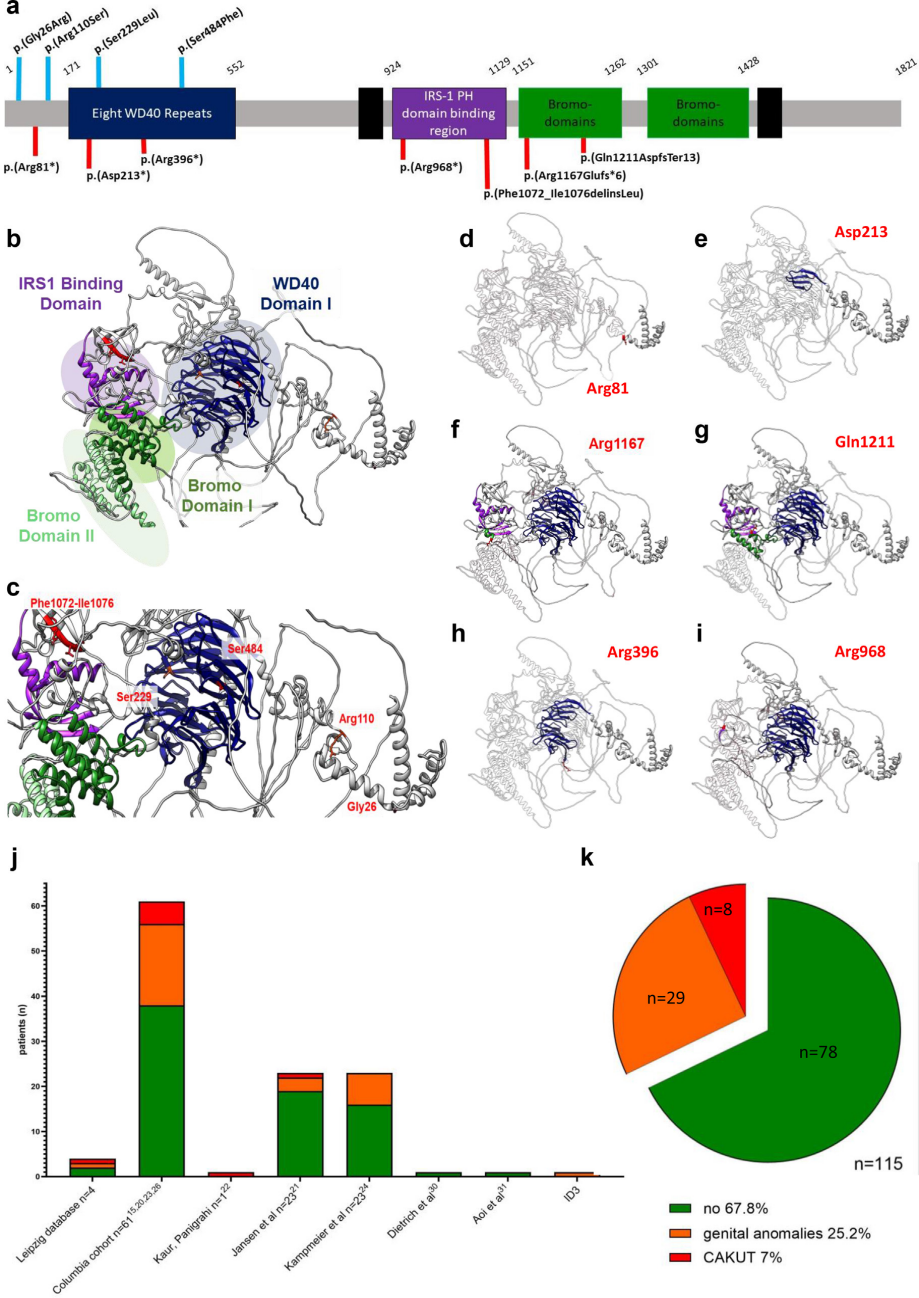
(a) 2D structure of *PHIP* with pathogenic variants: red poles indicate nonsense (truncating), blue poles denote missense variants. (b) 3D modelling with main functional domains (alpha-fold model Q8WWQ0-F1) [32]: intact 3D structure of PHIP, (c) missense variant residues, (d–i) nonsense variant residues with truncated c-terminal region shown as transparent. (j–k) Frequency of genital anomalies and CAKUT in patients with CHUJANS.

The ID1, a 42-year-old woman, underwent genetic testing before being placed on the kidney transplant waitlist. At the age of 40, the patient had developed kidney failure because of congenital bilateral hydronephrosis based on ureterovesical junction obstruction and recurrent urinary tract infections. In addition, the patient presented with dysmorphic facial features, obesity (Body mass index = 35.6 kg/m^2^), and infancy-onset developmental delay later evolving to intellectual disability (Figure 1a–e, Table 1). Next-generation sequencing-based gene-panel diagnostics did not detect pathogenic variants in established CAKUT genes or genes that were already associated with hereditary nephropathies. By exome sequencing, however, we detected a heterozygous nonsense variant (c.241C>T, p.Arg81∗) in *PHIP* leading to an abrogation of the eight β-propeller-forming WD40 repeat domain (Figure 2a, b, and d). This variant was found absent from population databases (gnomADv4) and is listed as pathogenic in ClinVar (Variation ID: 1710707).^28^ Parental segregation analysis revealed it to be a de novo variant (Table 1; Figure 1b).

**Table 1:** Patient characteristics

Patient	ID1	ID2.1	ID2.2	ID3	ID4^25^	ID5^25^	ID6^25^	ID7^25^	ID8^25^	ID9^22^	ID10^21^	ID11^19^
*PHIP* variant NM_017934.7												
c.pos.	c.241C>T	c.637_638del	c.637_638del	c.76G>A	c.3499del	c.3216_3227del	c.3628_3631delAAAC	c.1451C>T	c.1186C>T	c.2902C>T	c.328C>A	c.686C>T
p. pos.	p.Arg81^∗^	p.Asp213^∗^	p.Asp213	p.Gly26Arg	p.Arg1167 Glufs^∗^6	p.Phe1072_Ile1076delinsLeu	p.Gln1211Aspfs^∗^13	p.Ser484Phe	p.Arg396^∗^	p.Arg968^∗^	p.Arg110Ser	p.Ser229 Leu
CADD-PHRED	40	NA	NA	29.4	NA	NA	NA	33	NA	44	28.8	29.7
ClinVar	P	NA	NA	NA	P	LP	NA	LP	LP/P	LP/P	P	LP
Allele frequency (gnomADv4)	Not reported	Not reported	Not reported	Not reported	Not reported	Not reported	Not reported	Not reported	Not reported	Not reported	Not reported	Not reported
Sex	F	M	F	M	F	M	M	M	F	M	M	M
Inheritance	De novo	Maternal	Maternal	De novo	De novo	De novo	De novo	De novo	Unknown	NA	De novo	De novo
Age at inclusion (yr)	42	13	10	4	15	33	24	2.8	16.1	7	14	3
Age at 1st manifestation (yr)	Newborn	4	8 mo	Newborn	1.5	Newborn	24	Unknown	Unknown	3	1	Newborn
Urorenogenital phenotypes												
CKD (stage)^a^	G5d	G1	G1	G1	No data	No data	No data	No data	No data	G1	No data	No data
Age at KF (yr)	40	NA	NA	No	No	NA	No	NA	NA	NA	NA	No
Morphological kidney anomalies	No	Hypoplastic left kidney	NA	NA	Horseshoe kidney	Atrophic left kidney	Horseshoe kidney	Hydronephrosis	Unknown	Small right kid decr. cortico-medullary diff. & lobul-ated left kid	NA	NA
VUR	Yes	No	Severe III°	NA	No	No	No	Yes	Yes	NA	Yes	NA
PUV	No	No	NA	NA	No	No	No	Unknown	No	NA	NA	NA
Ureterocele	NA	No	NA	NA	No	No	No	No	No	NA	NA	Unilateral
UVJO	Bilateral	No	NA	NA	NA	No	No	No	No	NA	NA	NA
Incr. echogenicity	Yes	No	NA	No	NA	NA	NA	NA	No	No	NA	NA
Renal agenesis	No	No	NA	No	No	No	No	No	No	NA	NA	NA
Genital anomalies	No	No	No	Penoscrotal hypospadias, penis deviation, bilateral cryptorch.	No	No	Cryptorch, orchidopexy repair	Hypospadias	No	No	Genital hernia and frenulotomy	No
Other phenotypes												
Birth weight (low: yes/no)	NA	NA	NA	NA	No	NA	NA	NA	NA	No	Yes 2850 g mother HELLP	No
Height/percentile (cm)	165	NA	NA	98.6 (5th ct)	170	NA	NA	NA	NA	114	155	90
Body weight (kg)	100	NA	NA	12.5 (<1st ct)	128.8	NA	NA	NA	NA	39.2	51.5	13.7
BMI (kg/m^2^)	36.7	NA	NA	12.9 (1st ct)	44.6	NA	29.3	NA	34,8	30.2	22.7	16.9
Dysmorphic facial features	Yes	NA	NA	Yes	No	NA	NA	NA	NA	Yes	Yes	Yes
Short stature	NA	NA	NA	Yes	No	NA	NA	NA	NA	No	No	NA
ID DD	Yes	NA	NA	Yes	Mild	Yes	Yes	Yes	Yes	Yes	No	Yes
Behavioral problems	No	NA	NA	Anxiety, noise, sensitivity	ADHD, ODD, disruptive mood regul., anxiety, depression, psych. seizures	No	No	Impulsive behavior	Autism	Aggressive behavior, poor scholastic performance	Tics	No
Neurological problems	No	NA	NA	NA	Migraine	Hypotonia, motoric uncoor-dinated movement	Hypotonia, motoric uncoor-dinated movement	No	No	Hypotonia	No	Hypotonia, febrile seizures, red. white matter volume, cerebellar hypoplasia
Others	Recurrent UTIs	Syndactyly	Syndactyly pyelo-nephritis	Noise sensitivity, caries, VSD, constipation, talipes valgus, infections	PCOS, hearing loss/chronic ear infection, constipation	Strabismus	Astigmatism, diarrhea, scoliosis, pectus excavatum hearing loss	Astigmatism, diarrhea, recurrent UTI	GERD, constipation, recurrent UTIs	Recurrent UTIs hypo-thyroidism hip: decreased joint space bilaterally	Recurrent UTIs, recurrent ear infections	Recurrent UTI

ADHD, attention deficit hyperactivity disorder; BMI, body mass index; CKD, chronic kidney disease; cm, centimeter; comp. het., compound heterozygous; cryptorch, cryptorchidism; DD, developmental delay; GERD, gastroesophageal reflux disease; hom, homozygous; ID, individual; ID, Intellectual disability; KF, kidney failure; LP, likely pathogenic; mo, months; NA, not applicable (no data); ODD, oppositional defiant disorder; P, pathogenic; PCOS, polycystic ovary syndrome; pos, position; PUV, posterior urethral valve; UTIs, urinary tract infections; UVJO, ureterovesical junction obstruction; VSD, ventricular-septal defect; VUR, vesicouretral reflux; yr, years.

The variants reported refer to RefSeq NM_017934.7.

a Chronic kidney disease refers to the classification of the ‘Kidney Disease: Improving Global Outcomes’ (KDIGO) initiative. gnomAD v4 - population specific allele frequency (http://gnomad.broadinstitute.org/). CADD-PHRED scores were calculated using the following webtool: https://cadd.gs.washington.edu/snv.

For systematic evaluation of an association of PHIP haploinsufficiency and CAKUT, we clinically re-evaluated a large CHUJANS cohort (Columbia cohort), genetically assessed a large CAKUT cohort (Boston cohort), and used the GeneMatcher platform by searching for PHIP cases with CAKUT presentation.^24^ As a result, we identified 8 additional cases. Of note, ID4-8 were recently published as part of an aggregate description from the CHUJANS cohort at Columbia University.^25^ By searching the literature, we identified another 3 patients (ID9-11) with likely pathogenic/pathogenic *PHIP* variants and CAKUT.^15^^,^^19^, ^20^, ^21^, ^22^, ^23^^,^^29^^,^^30^ As a result, the frequency of urorenogenital phenotypes in patients with diagnostic *PHIP* variants and a clinical CHUJANS-diagnosis was found to range between 5% and 35% (*n* = 37/115) (Figure 2j and k), however, in patients with genetically unexplained CAKUT this was only the reason in 0.12% (*n* = 1/848).

### CAKUT-Cohort Evaluation

By trio exome sequencing of 848 families with genetically unresolved cases of CAKUT, we identified only 1 heterozygous nonsense variant in *PHIP* (c.637_638del, p.Asp213∗) in 2 siblings (ID2.1 and ID2.2). While the older sibling displayed a Prader-Willi-like syndrome with obesity and unilateral kidney hypoplasia, his younger brother showed severe unilateral ureteropelvic reflux resulting in recurrent pyelonephritis. Both presented with 2 of 3 toe syndactyly. The identified *PHIP* variant was inherited from the mother, who did not show any kidney or urogenital anomalies but did have syndactyly in common with ID2.1 and ID2.2 (Table 1; Figure 1f–k). The variant leads to a truncation within the 8 β-propeller-forming WD40 repeat domains (Figure 2a, b, and e).

### Matchmaker Initiatives

Another individual (ID3) was detected through GeneMatcher.^24^ The 4-year-old boy showed complex genitourethral malformations, specifically, a deep penoscrotal hypospadia, penis deviation, and bilateral cryptorchidism. He had corrective surgeries after birth, was growth retarded, and developmentally delayed with an anxiety disorder. Genetic testing revealed an ultrarare heterozygous missense variant in *PHIP* (c.76G>A, p.Gly26Arg) predicted to be deleterious by multiple in silico scoring systems (CADD-PHRED 29.4). The deduced amino acid change is located N-terminal of the 8 β-propeller-forming WD40 repeat domains. Parental segregation analysis confirmed a de novo status (Table 1; Figure 2a–c).

### CHUJANS-Cohort Evaluation

In the Columbia cohort, two patients (ID4 and ID6) had horseshoe kidneys with preserved kidney function. In both, molecular testing yielded *PHIP* frameshift variants (c.3499del, p.Arg1167Glufs∗6 and c.3628_3631delAAAC, p.Gln1211Aspfs∗13) affecting the N-terminal bromodomain. Similarly, ID5 carried a *PHIP* frameshift variant (c.3216_3227del, p.Phe1072_Ile1076delinsLeu) and displayed a congenitally atrophic left kidney (Table 1; Figure 2a–c, f, and g).^25^ Another 2 cases from this cohort had vesicoureteral reflux, 1 of them with hydronephrosis, associated with a deleterious missense variant (c.1451C>T, p.(Ser484Phe) (ID7) and a more C-terminal nonsense variant (c.1186C>T, p.Arg396∗) (ID8) (Table 1; Figure 2a–c and h).^25^ All variants were de novo, only in ID8, inheritance remained uncertain because of the lack of parental contact. More details of a part of this cohort were recently published.^25^

### Literature Research

Independently, systematic review of the literature for previously reported associations yielded the following 3 additional cases: (i) a 7-year-old boy (ID9) with abnormal weight gain, aggressive behavior, dysmorphic facial features, and hypothyroidism.^22^ On performing kidney ultrasound, he exhibited a lobulated left kidney and a hypoplastic right kidney with decreased corticomedullary differentiation. Additionally, the medical record noted recurrent urinary tract infections. The clinical syndrome was associated with loss of function of *PHIP* owing to a premature termination of the protein (c.2902C>T, p.Arg968∗); pathogenic in ClinVar, ID: 523846) within the insulin receptor subtrate-1 pleckstrin homology domain binding region (Table 1; Figure 2a, b, and i).^22^ (ii) A 14-year-old boy (ID10) with tics, mild hip dysplasia, and dysmorphic facial features, who had recurrent urinary tract infections owing to vesicoureteral reflux and required frenulotomy.^21^ Genetic testing yielded a de novo missense variant in *PHIP* (c.328C>A, p.Arg110Ser); pathogenic in ClinVar, ID: 545397, CADD-PHRED 28.8), which resides N-terminal of the 8 β-propeller-forming WD40 repeat domains (Table 1; Figure 2a–c). (iii) Lastly, a 3-year-old boy (ID11) with unilateral ureterocele, recurrent urinary tract infections, muscular torticollis, sacro-coccygeal dimple, generalized hypotonia, and dysmorphic facial features.^19^ Development was delayed, and there was reduced white matter and cerebellar hypotrophy on brain MRI.^19^ Molecular diagnostics demonstrated a heterozygous, likely pathogenic de novo missense variant in *PHIP* (c.686C>T, p.Ser229Leu); likely pathogenic in ClinVar, ID: 627528, CADD-PHRED 29.7)^19^ also effecting the WD40 repeat domains (Table 1, Figure 2a–c).

On the protein level, all 4 missense variants are located in the highly conserved N-terminus (Figure 2c). Similarly, 3 out of 7 truncating alterations are located N-terminal or within the 8 β-propeller-forming WD40 repeat domains. We applied alpha-fold protein structure database to predict the impact on 3D structure (3D model alphafold (Q8WWQ0-F1) (Figure 2b–i).^31^

### PHIP Exhibits Strong Expression in Normal Human Embryonic Kidneys

**Figure 3:**
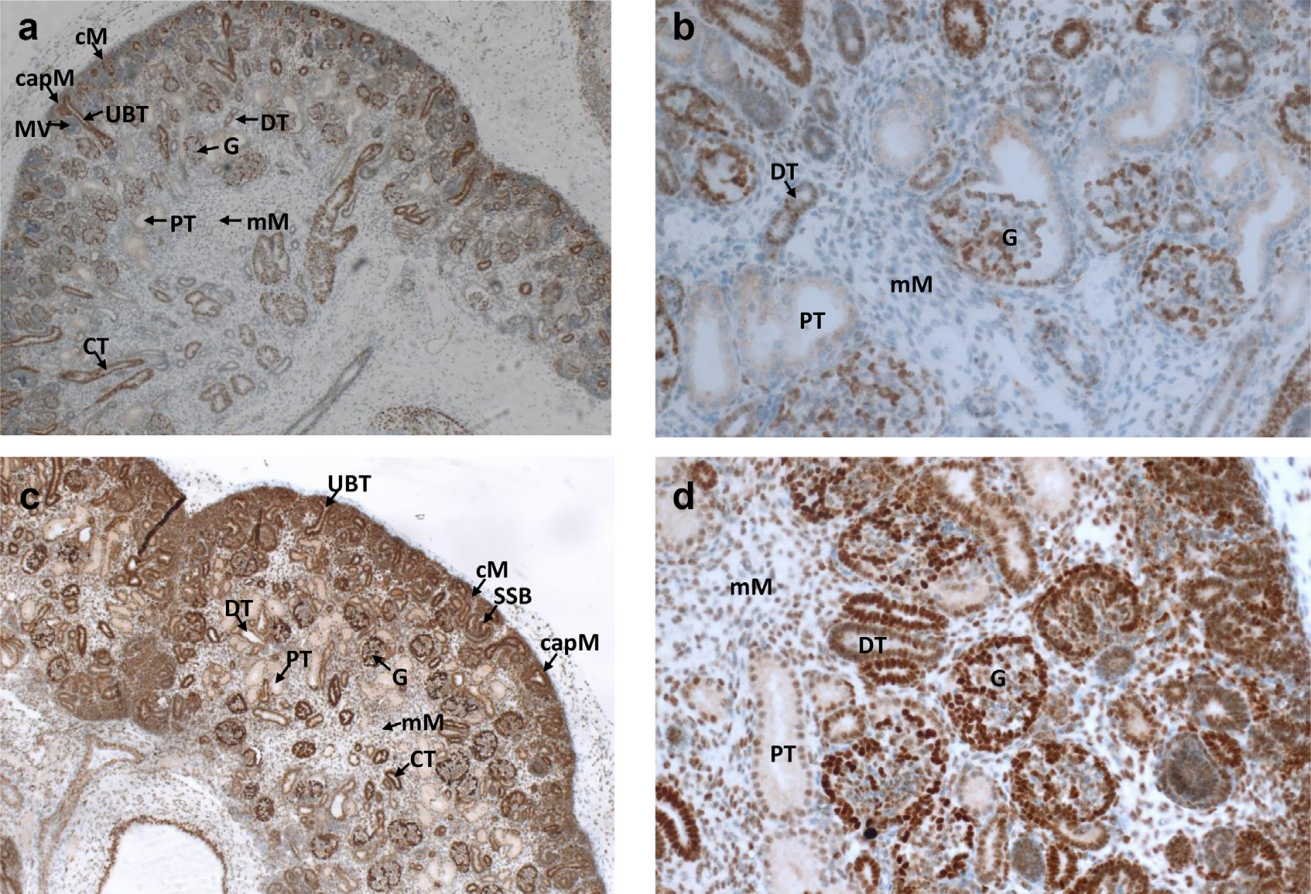
Immunohistochemical staining targeting PHIP in human embryonic control kidneys. (a–b) 14 weeks of gestational age, (c–d) 22 weeks of gestational age. cM, Cortical mesenchyme; CapM, Cap mesenchyme; CT, Collecting tubule; DT, Distal tubule; G, Glomerulus; mM, Medullary mesenchyme; MV, Metanephric vesicle; PT, Proximal tubule; UBT, Uteric bud tip.

To address the question whether PHIP-defects may be causally related to CAKUT, we investigated PHIP expression in human fetal kidneys from unaffected controls. Staining of human kidney sections from the 14^th^ and 22^nd^ week of gestation using a polyclonal PHIP antibody revealed widespread expression in the developing kidney. Strong expression was observed in the collecting tubules and the ureteric bud tips, glomerular cells, and the cortical mesenchyme. Moderate expression was found in the cap mesenchyme and the distal tubules, with very low expression in metanephric vesicles, s-shaped bodies, the medullary mesenchyme, and proximal tubules (Figure 3a–d).

### Robust Phip Expression was Observed in Murine Embryonic Kidneys

**Figure 4:**
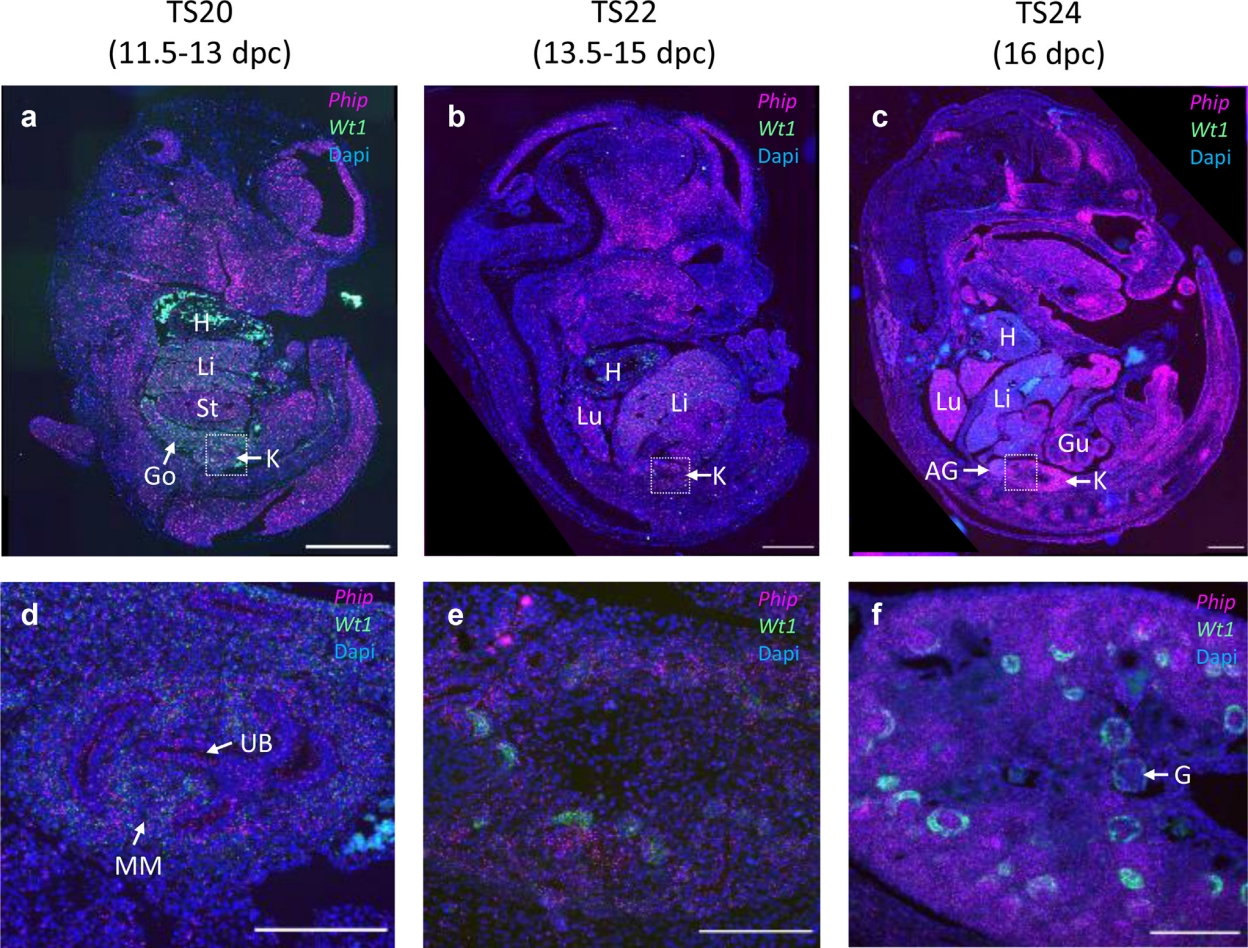
RNAScope in situ hybridization of Phip (pink) was conducted on mouse embryos at different developmental stages (a: TS20, b: TS22 and c: TS 24). Wt1 (green) was used as a marker for mesenchymal cells, comma/s-shaped bodies, and glomeruli in the developing kidney. Dapi (blue) was used for visualization of nuclei. (a–c) longitudinal section of the whole embryo. (d–f) Magnification of the developing mouse kidney from the dotted squares in (a–c). Scale bar in upper panels indicates 200 μm and in lower panels 1000 μm. Note: Unspecific green staining of erythrocytes in the heart and large blood vessels. AG, Adrenal gland; Dapi, 4’,6-diamidino-2-phenylindole; Dpc, Days post conceptionem; G, Glomerulus; Go, Gonad; Gu, Gut; H, Heart; K, Kidney; Li, Liver; Lu, Lung; MM, Metanephric mesenchyme; Phip, Pleckstrin homology domain-interacting protein; St, Stomach; TS, Teiler stage; UB, Uteric bud; Wt1, Wilms tumor 1.

To further investigate PHIP organ expression patterns during embryonic and fetal development, we investigated murine tissues from various developmental stages. Embryonic and postnatal in situ hybridization using RNAscope provided an overview of Phip expression during organogenesis (Figure 4). In early embryogenesis (TS 20) Phip RNA was expressed in most tissues, including gonad and kidney (Figure 4a). The expression increased at TS 22 in tissues with high mitotic activity, such as precursor thalamus or diencephalon (Figure 4b). During later stages of embryogenesis (TS 24), PHIP expression became more pronounced, with strong emphasis on selected organs, including the developing kidney (Figure 4c). In the early embryonic mouse kidney (TS 20), PHIP expression was localized to both the mesenchyme and the ureteric bud (Figure 4d). At TS 24 Phip expression was evident in all structures of the cortical region (Figure 4f). However, glomerular Phip expression was low.

### Renal Phip Expression Decreases Postnatally

**Figure 5:**
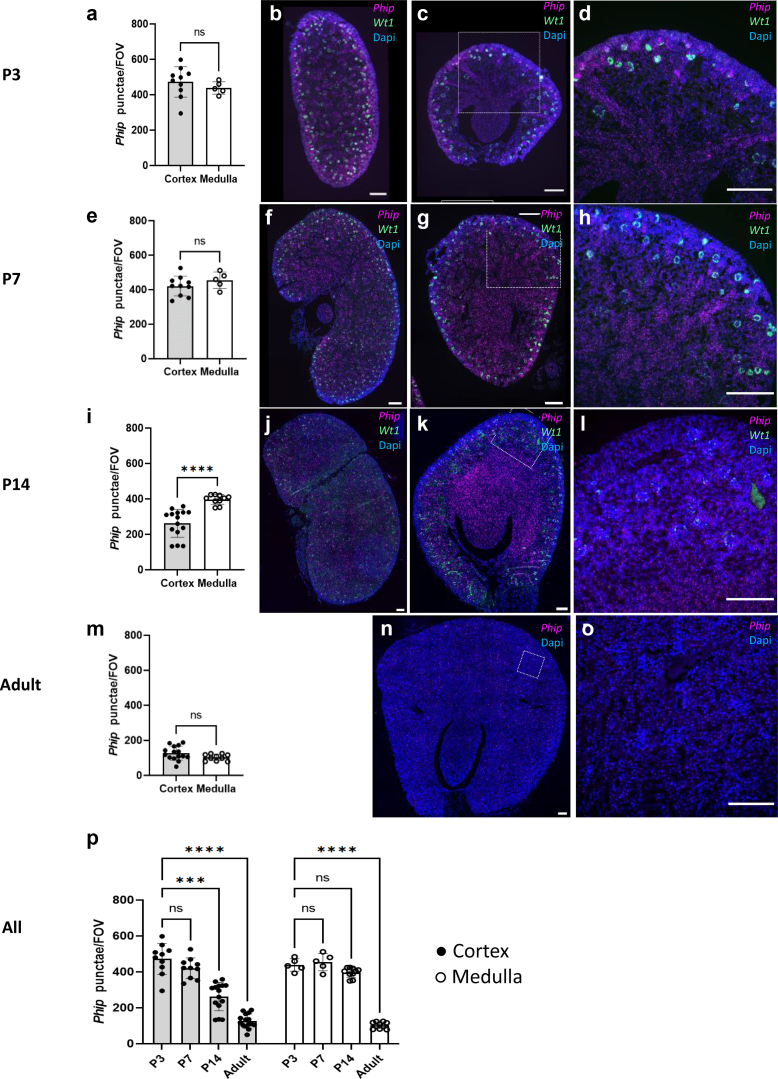
RNAScope in situ hybridization and quantification of Phip expression of postnatal mouse kidneys at different developmental times (a–d) 3 days postnatal, (e–h) 7 days postnatal, (i–l) 14 days postnatal, (m–o) adult (11 weeks), (p) all stages-bar graph summary stastics. Wt1 (green) was used as a marker for glomeruli. Dapi (blue) was used for visualization of nuclei. Dotted squares represent the area from which images c, g, k, and n were acquired respectively. Scale bars represent 200 μm.

To investigate Phip expression postnatally, we performed RNA in situ hybridization in murine kidney sections. At developmental stage P3 Phip expression was most prominent in the outer cortical regions and in the collecting ducts (Figure 5b–d). As kidney development progressed, expression in the outer cortical activity regions declined at P7 (Figure 5f–h), followed by a further decrease at P14 (Figure 5j–l) and disappeared in the kidneys of adult mice (Figure 5n, and o). Overall, the expression of Phip persisted in the medullary region at P7 and P14 (Figure 5g and k) and was lost in the kidneys of adult mice. Quantification of renal Phip expression (Figure 5a, e, and i) indicated equal expression in the cortical and medullar regions at P3 and P7, with a significant decline in cortical PHIP expression at P14 and a generally low Phip expression in the adult kidneys (Figure 5m and p).

## Discussion

In this study, we report on congenital urorenogenital anomalies as part of the CHUJANS-spectrum by identification and characterization of 4 individuals (ID1-ID3) with (likely) pathogenic *PHIP* variants. Additionally, we found 5 individuals from a large PHIP cohort (ID4-8) and 3 individuals with CHUJANS and syndromic CAKUT through systematic literature review (ID9-ID11).^19^^,^^21^^,^^22^^,^^25^ When interrogating CHUJANS cohorts retrospectively, we found kidney and urogenital phenotyping to be underrepresented, leaving the possibility of undiagnosed CAKUT in CHUJANS. Therefore, we believe that ∼5% to 35% of individuals with CHUJANS exhibit CAKUT and/or genital anomalies as a variable feature of the phenotypic spectrum.

CAKUT represents one of the most common malformations at birth^32^ and is a common cause for kidney failure over lifetime.^2^^,^^4^ In the majority of cases, the underlying cause remains unknown,^7^^,^^33^^,^^34^ probably because of genetic heterogeneity, variable expressivity, incomplete penetrance, and environmental contributors.^2^ Many CAKUT genes are associated with multiorgan dysfunction, resulting in a large number of syndromic cases including neurodevelopmental disorders.^2^^,^^35^, ^36^, ^37^, ^38^, ^39^ Based on this knowledge, further investigation of patients with known congenital syndromes might enable the discovery of novel candidate genes for CAKUT.^9^, ^10^, ^11^

The full-length protein complex CUL4-DDB1 (DCAF14) encoded by *PHIP*^13^^,^^14^ forms the active substrate receptor of E3 ubiquitin ligases.^16^ It controls posttranslational modifications of many regulators of the cell cycle and DNA replication and might, therefore, be active in all tissues with high mitotic activity.^17^^,^^18^^,^^40^, ^41^, ^42^ Furthermore, DCAF14 enhances replication fork stability by preventing fork collapse into toxic double-strand breaks protecting DNA from degeneration and thus protecting genome integrity.^15^^,^^40^ These crucial regulatory functions of PHIP in the cell cycle make it very likely that haploinsufficiency has systemic effects. Phenotypic changes can, therefore, be expected in many different tissues, including the urorenogenital tract. Previously, only cryptorchidism was part of the known phenotypic spectrum of CHUJANS,^19^^,^^21^^,^^23^ indicating a role in urogenital development in humans. Of note, a large number of DCAFs, which are conserved WD40 genes, are highly expressed during testicular development and spermatogenesis in mice and humans.^41^

The murine RNA in situ hybridization data show that PHIP localizes to diverse developing organs, such as the central nervous system, lungs, and the kidneys. These high levels of physiological PHIP expression in the kidneys increase the possibility of causing CAKUT when falling below a certain threshold. The postnatal decline in PHIP expression levels provides additional evidence supporting this assumption and is almost undetectable in the fully developed kidneys of adult mice. This is in accordance to other findings of expression pattern of DCAFs at different developmental stages of the testis.^41^ In a transgenic mouse model of PHIP deficiency, decreased length and decreased overall survival were observed, supporting a role in postnatal growth and lifespan.^43^

Human single-cell transcriptomic databases underscore the role of PHIP during nephrogenesis with high expression in nephron progenitor, interstitial proliferating, and tubular precursor cells at the 17^th^ week of gestation (http://humphreyslab.com/SingleCell/displaycharts.php).^44^^,^^45^ Differences in cortical and medullar PHIP expression at different development stages might illustrate the variable tissue differentiation at 1 time point. In the medulla, there are regions with enhanced PHIP signaling, which could correspond to yet undifferentiated mesenchymal progenitor cells.

Interestingly, urorenal malformations were extremely heterogeneous, ranging from a horseshoe kidney to kidney hypo/dysplasia, to vesicoureteral reflux. Although ID3 did not exhibit classical CAKUT features, it is worth noting that the lower urethra develops from the same cloacal endoderm-derived tissue as most parts of the bladder. This is in contrast to the mesoderm-driven metanephros, which likely explains the presence of a shortened urethra and penoscrotal hypospadias.^46^ Disruption of the interaction between endoderm and mesoderm during urogenital development or deficient ureter budding might result in a defective connection of the ureter to the bladder leading to vesicoureteral reflux.^47^

Immunohistochemistry targeting PHIP in human embryonic kidneys indicated high levels of DCAF14 in the nephrogenic zone of the cortex, which has high proliferative activity (Figure 3). This finding supports the regulatory function of DCAF14 in sustaining genome integrity of newly synthesized DNA in tissues with high mitotic activity.^40^

Furthermore, PHIP shows high expression in tubular progenitor structures (Figures 3 and 4), aligning with the E3 ubiquitin ligases regulated expression of tubular transporters, such as Na^+^/K^+^/2Cl^−^ cotransporter or thiazide-sensitive sodium chloride cotransporter.^48^^,^^49^

All reported missense variants and the half of the truncating variants are located N-terminal or within the 8 β-propeller-forming WD40 repeat domains, which mediate binding of ubiquitin ligase component CUL4 to chromatin before DNA replication. N-terminal or active domain changes result in CUL4/DDB1 ubiquitin ligase dysfunction and substrate accumulation. This leads to genomic instability with double-strand breaks and dysregulation of the cell cycle.^42^ However, changes in this region are not specific to urorenogenital involvement and also appear to be common in CHUJANS-patients without CAKUT.^19^, ^20^, ^21^^,^^23^^,^^25^

In summary, this study reports on patients with CHUJANS and concomitant CAKUT carrying deleterious *PHIP* variants. Based on high embryonic expression shown on both RNA and protein level, a role of PHIP during embryonic kidney development seems plausible, however we cannot demonstrate causality. This assumption supports the function of PHIP as an important regulator of the cell cycle and replication, especially during organogenesis. However, CAKUT does not occur in most patients with CHUJANS, suggesting variable expressivity that may involve additional genetic or epigenetic factors triggering kidney development impairment.

Conclusively, for patients who were undiagnosed with syndromic CAKUT, which includes neurodevelopmental delay or intellectual disability and obesity, PHIP defects should be considered as part of the differential diagnosis. Conversely, patients diagnosed with CHUJANS should undergo assessment for kidney and urogenital anomalies.

One of the major challenges in complex congenital anomaly syndromes lies in comprehensive phenotyping across various medical disciplines. Because phenotypic characterization often prioritizes the initially affected organ system, there is an urgent need for more comprehensive and systematic organ assessment. Adequate description of multiorgan involvement will be facilitated by digital phenotyping tools that enable systematic and structured data capture in the future.^50^

## Disclosure

All the authors declared no competing interests.

## Patient Consent

The study was approved by the Institutional Review Board (IRB) at the University of Leipzig, Germany (IRB00001750; #402/16-ek) and the IRBs at Columbia University and Boston Children´s Hospital. Written informed consent to participate in this study was provided by the patients or the participant’s legal guardian.
